# Simultaneous Sodium and Calcium Imaging from Dendrites and Axons

**DOI:** 10.1523/ENEURO.0092-15.2015

**Published:** 2015-11-09

**Authors:** Kenichi Miyazaki, William N. Ross

**Affiliations:** 1 Department of Physiology, New York Medical College, Valhalla, New York 10595; 2 Marine Biological Laboratory, Woods Hole, Massachusetts 02543

**Keywords:** calcium, imaging, sodium

## Abstract

Dynamic calcium imaging is a major technique of neuroscientists. It can reveal information about the location of various calcium channels and calcium permeable receptors, the time course, magnitude, and location of intracellular calcium concentration ([Ca^2+^]_i_) changes, and indirectly, the occurrence of action potentials. Dynamic sodium imaging, a less exploited technique, can reveal analogous information related to sodium signaling. In some cases, like the examination of AMPA and NMDA receptor signaling, measurements of both [Ca^2+^]_i_ and [Na^+^]_i_ changes in the same preparation may provide more information than separate measurements. To this end, we developed a technique to simultaneously measure both signals at high speed and sufficient sensitivity to detect localized physiologic events. This approach has advantages over sequential imaging because the preparation may not respond identically in different trials. We designed custom dichroic and emission filters to allow the separate detection of the fluorescence of sodium and calcium indicators loaded together into a single neuron in a brain slice from the hippocampus of Sprague-Dawley rats. We then used high-intensity light emitting diodes (LEDs) to alternately excite the two indicators at the appropriate wavelengths. These pulses were synchronized with the frames of a CCD camera running at 500 Hz. Software then separated the data streams to provide independent sodium and calcium signals. With this system we could detect [Ca^2+^]_i_ and [Na^+^]_i_ changes from single action potentials in axons and synaptically evoked signals in dendrites, both with submicron resolution and a good signal-to-noise ratio (S/N).

## Significance Statement

Dynamic imaging is an important technique of neuroscientists. It can reveal information about the location and activation of various channels and receptors, the time course, magnitude, and location of intracellular ion concentration changes, and, indirectly, the occurrence of action potentials. Most experiments exploit [Ca^2+^]_i_ changes, but measurements of [Na^+^]_i_ changes, a less exploited technique, can reveal analogous information related to sodium signaling. We developed a technique to measure both signals simultaneously at high speed and sensitivity from localized regions of individual neurons in brain slices. With this method, we can measure and analyze aspects of dendritic and axonal physiology that could not easily be determined from measurements of sodium or calcium changes separately.

## Introduction

Calcium imaging has been an important tool of neuroscientists and other physiologists for >25 years. It can reveal information about the location and activation of various calcium channels and calcium permeable receptors, the time course, magnitude, and location of intracellular calcium concentration ([Ca^2+^]_i_) changes, and indirectly, the occurrence of action potentials. Dynamic sodium imaging, a less exploited technique, can reveal analogous information related to sodium signaling. Sodium imaging has been used to show the location of sodium channels in the axon (Fleidervish et al., 2010) and dendrites (Callaway and Ross, 1997) of cerebellar Purkinje neurons and pyramidal neurons (Jaffe et al., 1992; Rose et al., 1999), and the activation of glutamate receptors on these same cells (Callaway and Ross, 1997; Knöpfel et al., 2000; Scelfo et al., 2003). This approach is particularly relevant for AMPA receptor signaling because these receptors are not calcium permeable in many neurons. Also, there are some signaling pathways that are activated by changes in [Na^+^]_i_ (Bhattacharjee and Kaczmarek, 2005), and these might be illuminated by sodium imaging experiments. Even with these opportunities, however, there have been few papers based on this approach.

Most dynamic sodium imaging experiments in brain slices have used standard fluorescence imaging technologies, ie, an arc lamp epifluorescence light source, a filter cube matched to the indicator, and a CCD camera to detect the change in fluorescence. Only a few experiments have used two-photon microscopy to examine [Na^+^]_i_ changes in neurons (Rose et al., 1999; Rose and Konnerth, 2001), possibly because the signals are weak and single spine signals, which would exploit this technology, have not been reported (see Discussion). In addition, there has been little effort to develop a range of sodium indicators with different sensitivities and spectral properties, which could be used in different kinds of experiments. The original indicator, sodium-binding benzofuran isophthalate (SBFI), synthesized by Minta and Tsien (1989), is still the most commonly used indicator. Newer indicators, such as CoroNa green, have not been found adequate in experimental conditions (Meier et al., 2006).

Starting from this perspective, we tried to improve the sensitivity and spatial resolution of sodium signals. We found that high-intensity light emitting diode (LED) light sources, a low-noise, high-speed CCD camera, and high numerical aperture objectives all improved the signals (see Discussion). In addition, a new indicator, Asante NaTRIUM Green-2 (ANG-2, Teflabs) was found to be better than SBFI in some conditions. As we implemented these improvements, we found that they permitted the design of a system that allowed simultaneous sodium and calcium imaging. Simultaneous imaging has advantages over sequential imaging using two different filter cubes because the preparation may not respond identically in different trials, for example, if there are synaptic failures in experiments measuring responses from AMPA and NMDA receptor activation. Our system uses custom dichroic and emission filters to separately excite sodium and calcium indicators loaded into the same cell. Quasi-simultaneous detection of the fluorescence signals was effected by alternately illuminating each indicator at 2 ms intervals. Signals from each indicator were then detected in successive frames of the CCD camera operating at 500 Hz. Software was then used to separate the signals into two parallel data streams. Tests showed that these procedures did not introduce additional noise. The signal-to-noise ratio (S/N) of the system was still limited by shot noise (stochastic fluctuations in the number of detected photons).

## Materials and Methods

### Indicators

**Figure 1.**
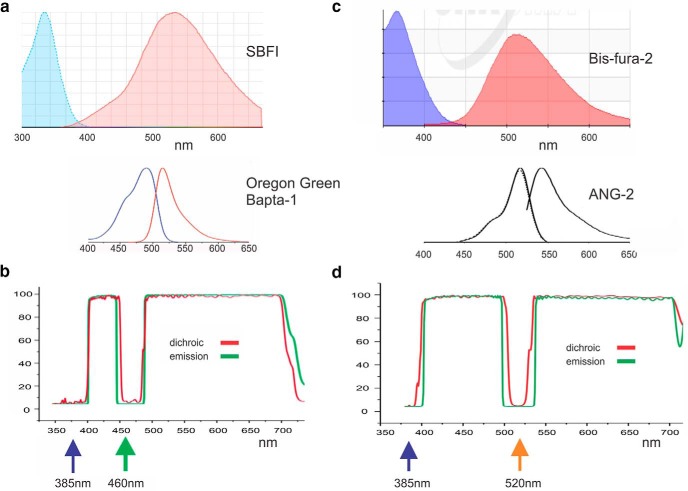
Design of dichroic and emission filters for the simultaneous detection of sodium and calcium changes. ***a***, Excitation and emission spectra of SBFI and OGB-1 in cuvettes (from the Chroma Technology website). The spectra have been arranged so the same wavelengths are aligned vertically. The excitation spectrum of SBFI shows the sodium bound form. The spectra of OGB-1 are much narrower. ***b***, Design of custom dichroic and emission filters to detect responses from these indicators. The dichroic cuts on at 390 nm to allow excitation of SBFI at 380 nm. The notch at 460 allows excitation of OGB-1, which has a significant shoulder at that wavelength. The emission filter passes most of the emitted fluorescence from both indicators, missing only a part of the SBFI fluorescence around 460 nm. ***c***, Spectra of bis-fura-2 and ANG-2 (from the Molecular Probes website and Roder and Hille, 2014). ***d***, Design of custom dichroic and emission filters to detect responses from these indicators.

To simultaneously detect sodium and calcium signals, we chose indicators that could be separately excited with illumination at different wavelengths (Fig. 1*a*,*c*). One sodium indicator, SBFI, is optimally excited at 370 nm, although the excitation peak appears to be quite broad (Birkedal and Shiels, 2007), allowing excitation at 385 nm, the peak of the LED spectrum. We paired SBFI with calcium indicators in the Oregon Green series, either OGB-1 (Oregon Green BAPTA-1) or OGB-5N (Oregon Green BAPTA-5N). Tests showed that OGB-1 was slightly excited at the sodium LED peak of 385 nm (∼2% of excitation at 460 nm). This crosstalk was ignored. If necessary, computational algorithms can correct for this effect. The second sodium indicator, ANG-2 (Lamy and Chatton, 2011; Schreiner and Rose, 2012; Roder and Hille, 2014), is optimally excited at 520 nm. We paired this indicator with bis-fura-2 and other indicators in the fura series that can be excited at 385 nm.

### Optical filters

Switching between two excitation bands is related to the way fura-2 is excited at 340 and 380 nm to activate the calcium-bound and calcium-free forms of that compound. However, the emission bands of these two forms of fura-2 are the same and are shifted to the red allowing a single dichroic mirror and emission filter to be used with either excitation band. The spectral properties of neither of the sodium/calcium indicator combinations we used allow for this simple combination; the excitation band of the longer wavelength indicator overlaps significantly with the emission band of the shorter wavelength indicator (Fig. 1*a*,*c*). Therefore, we used a different approach.

We designed custom dichroic and emission filters (Chroma) that allow excitation of the longer wavelength indicator while blocking only a small part of the emission spectrum of the shorter wavelength indicator (Fig. 1*b*,*d*). For the SBFI/OGB-1 combination, the dichroic mirror had a notch ∼20 nm wide centered at 460 nm. This wavelength matched the available 460 nm LED. Although 460 nm is not the peak of OGB-1 excitation (it is 488 nm), there was sufficient excitation of this indicator that detecting OGB-1 emission light was not a limiting factor in our experiments. Furthermore, the notch at 460 nm blocks less of the SBFI emitted light than a notch at 488 nm would block (Fig. 1*a*). In retrospect, we might have designed the filters with narrower notches, allowing more emission light from SBFI through to the detector. For the bisfura-2/ANG-2 combination, we designed similar filters with a notch centered at 520 nm (Fig. 1*d*).

### LED light sources

**Figure 2.**
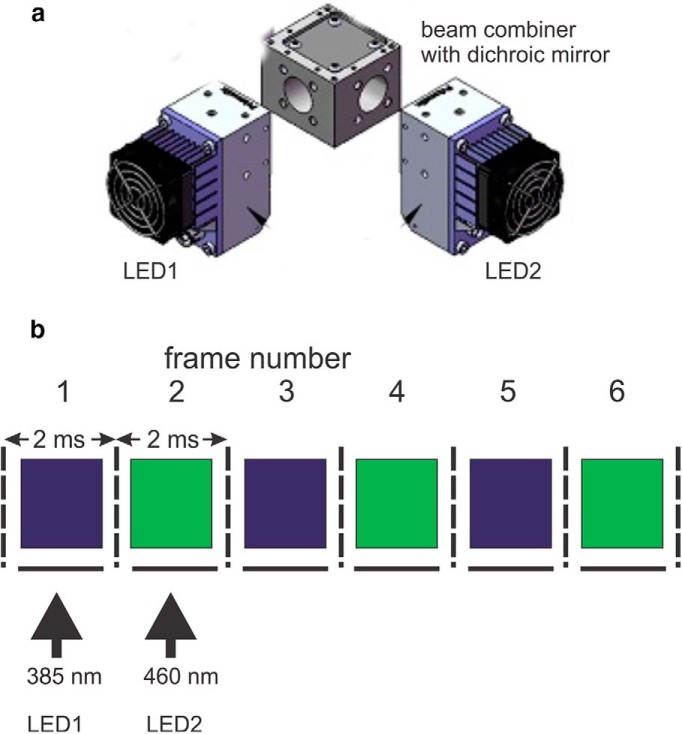
***a***, Arrangement of LEDs at the excitation port of the microscope fluorescence illuminator. The outputs of the two LEDs are combined with a dichroic mirror centered at 425 nm. ***b***, Spectra of LEDs used in our experiments (from Prizmatix catalogue). They are nominally centered at the indicated wavelengths but each emits some light over a 50–100 nm range. Below the spectra are the bandpass ranges of excitation filters used to limit the excitation light. The sequence of frame illumination in a typical experiment; at 500 Hz each frame has duration of 2 ms (dotted vertical lines). The LED turns on 0.1 ms after the start of the frame and turns off 0.1 ms before the end of the frame. In the next frame, the second LED is on for the same duration. This alternating sequence continues for the duration of the trial. A similar arrangement was used when pairing the 385 and 520 nm LEDs.

We selected high-power LEDS from Prizmatix for these experiments. These LEDs can be combined using appropriate dichroic mirrors so that different pairs illuminate the fluorescence port of the microscope (Fig. 2*a*). Similar LEDs are available from other suppliers, but not always with the same peak wavelengths or power. We placed narrow excitation filters (380/15 for SBFI; 387/11 for bis-fura-2; 470/22 for OGB-1; 520/15 for ANG-2) in front of the individual LEDs (2 are shown in Fig. 2*b*). These filters removed the skirts from the spectra of the LEDs, which are not monochromatic. To assay the intensity of the LEDs we compared their light output through these filters with the light output of a standard 75W Xenon arc lamp in an Olympus lamp housing. We found that the three LEDs were 8.5 (385 nm), 13.5 (460 nm), and 8.7 (520 nm) times as bright as the output of the arc lamp source with the same excitation filters. These intensities were more than sufficient for our experiments. In fact, it was often necessary to lower the LED outputs to reduce photodynamic damage to the preparation.

### Quasi-simultaneous imaging

One advantage of LED light sources is that they can be turned on and off in microseconds. Therefore, fast shutters, which sometimes cause vibrations, are not necessary with these devices. We used this property to alternatively activate each LED at ∼2 ms intervals; the exact time was adjusted to match the frame rate of the NeuroCCD-SMQ camera (RedShirtImaging), which was nominally set at 500 Hz but actually was at 500.80 Hz. The timing pulse protocol was determined by a short Labview program controlling a National Instruments NI PCIe-6320 board in a computer. These timing pulses drove variable width pulsers, which in turn activated the LEDs (Fig. 2*b*). The pulse delay and width were set so as not to overlap the time when the CCD camera was reading out data from the CCD chip (∼100 µs). With this protocol, we confirmed that there was no crosstalk between frames illuminated with the two different LEDs by turning off one LED during a trace and finding that there was no change in the signal from the channel with the other LED.

### Slice preparation and electrophysiological procedures

Most experiments used hippocampal slices from 2- to 4-week-old Sprague-Dawley rats of either sex prepared using protocols standard for our laboratory. All procedures were approved by institutional IACUC committees. Submerged slices were placed in a chamber mounted on a stage rigidly bolted to an air table and were viewed with water-immersion lenses in an Olympus BX50WI microscope mounted on an *X–Y* translation stage. For maximum light detection and spatial resolution, we used an Olympus 60X, 1.1 NA lens. Even with this high NA, it was possible to patch neurons under visual control. In other experiments, we used 20× or 40× lenses, which have longer working distances and lower NAs. Slices were superfused at ∼1 ml/min with standard ACSF consisting of the following (in mM): 124 NaCl, 2.5 KCl, 2 CaCl_2_, 2 MgCl_2_, 1.25 NaH_2_PO_4_, 26 NaHCO_3_, and 10.1 glucose. Somatic whole-cell recordings were made using patch pipettes pulled from 1.5 mm outer diameter thick-walled glass tubing (1511-M, Friedrich & Dimmock). Tight seals on CA1 pyramidal cell somata were made with the “blow and seal” technique using video-enhanced DIC optics to visualize the cells (Stuart et al., 1993) For most experiments the pipette solution contained the following (mM): 130 potassium gluconate, 4 Mg-ATP, 0.3 Na-GTP Tris salt, and 10 Hepes, 7 potassium phosphocreatine, pH adjusted to 7.3 with KOH. This solution was supplemented with indicator combinations at concentrations from 0.05 to 2 mM (see Results).

### Data taking and analysis

Experiments were under the control of Neuroplex software, which came with the RedShirtImaging camera. This program determined the frame rate of the camera, synchronized the recording of electrical and optical signals, controlled the initiation of the LED pulse sequence, and triggered a Master-8 pulser, which in turn controlled the timing and duration of intrasomatic pulses and activated a synaptic stimulation protocol in some experiments. The resulting optical and electrical data were then processed through two custom programs written in our laboratory. The first transformed the data into a format more easily read by other programs and split the two interdigitated channels into two separate data streams. The second program, SCAN, performed all the data analysis. New routines were written to allow the easy comparison of the sodium and calcium signals from the same locations. These programs are available from the authors. Finally, in some experiments we noticed that small vibrations or movements distorted the optical signals from small regions-of-interest (ROIs) next to the edge of the axon or dendrite. These movements were corrected by a program that shifted each frame to achieve maximum registration with the first frame of the sequence. It used a standard algorithm that implemented nonlinear optimization and matrix-multiply discrete Fourier transforms (Guizar-Sicairos et al., 2008) and was coded in MATLAB.

## Results

**Figure 3.**
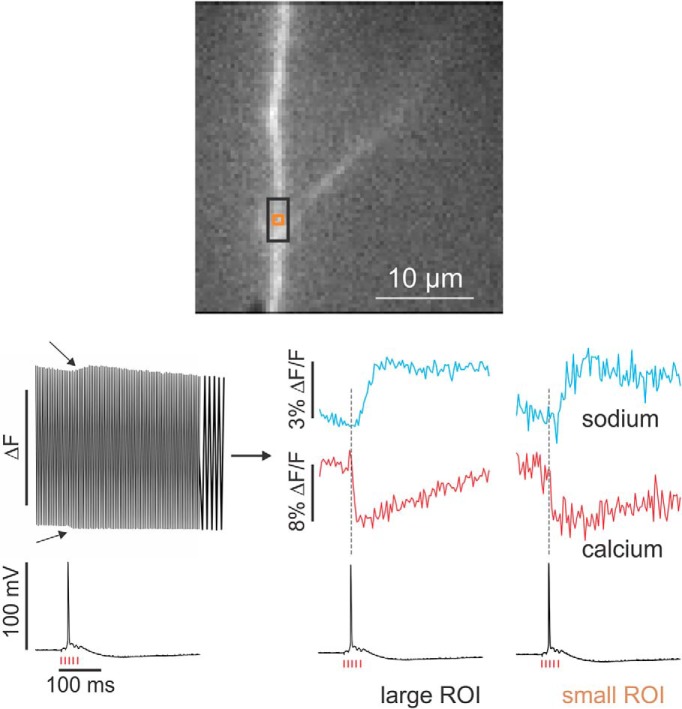
Simultaneous sodium and calcium imaging from an apical dendrite of a hippocampal CA1 pyramidal neuron. The image shows a small region of the apical dendrites (32 × 32 µm^2^). The fluorescence is from ANG-2 injected from a patch electrode on the soma. The black ROI is ∼3 × 5 µm^2^; the smaller orange ROI is ∼0.8 × 0.8 µm^2^. Five pulses at 100 Hz from a stimulating electrode on the SC near the ROI generated a synaptic response that evoked a single AP. The optical recording on the left shows the fluorescence levels in the black ROI when the cell was alternately illuminated at 500 Hz at 385 and 520 nm. The last 20 ms are stretched out to show the alternating response more clearly. There is an envelope to the signals at the top and bottom of the recording (arrows). The traces on the right show the records when the two channels are separated. Traces were corrected for bleaching (see Discussion). There are clear signals in both the sodium and calcium channel. Note that there is a delay in the onset of the sodium signal compared to the spike time, whereas the calcium signal is synchronous with the spike. This is clearer in the black ROI, which has less noise because it integrates over a larger area. The calcium signal goes down because bis-fura-2 fluorescence decreases when [Ca^2+^]_i_ increases.

In a typical experiment, we patched the soma of a CA1 pyramidal neuron with an electrode containing 200 µM bis-fura-2 and 200 µM ANG-2. After allowing ∼30 min for the indicators to diffuse into the dendrites we stimulated the Schaffer collaterals with a brief tetanus, evoking a synaptic response recorded in the somatic electrode. Figure 3 (left) shows the raw optical response detected by the camera at a single site in the dendrites (black ROI). With each frame, the signal oscillates back and forth between the fluorescence level evoked by the 385 and 520 nm LEDs. It is also clear that there is a slow change on both the top and the bottom of this signal (Fig. 3, arrows). These correspond to the fluorescence changes generated by the sodium and calcium changes at that site. Figure 3 (right) shows the signals in the separate channels in the same ROI (black) and in a smaller region (orange ROI). The signals go in opposite directions because ANG-2 fluorescence increases when [Na^+^]_i_ increases, whereas bis-fura-2 fluorescence decreases when [Ca^2+^]_i_ increases. The signals are different (the calcium signal occurs at the time of the spike but the sodium signal is delayed) but both are aligned with the synaptic electrical response. This is more obvious in the less noisy traces from the larger black ROI. The clear signals from the orange ROI (Fig. 3, right) show that this system can simultaneously detect both sodium and calcium signals from synaptic events from submicron sized regions without temporal averaging and at high time resolution.

**Figure 4.**
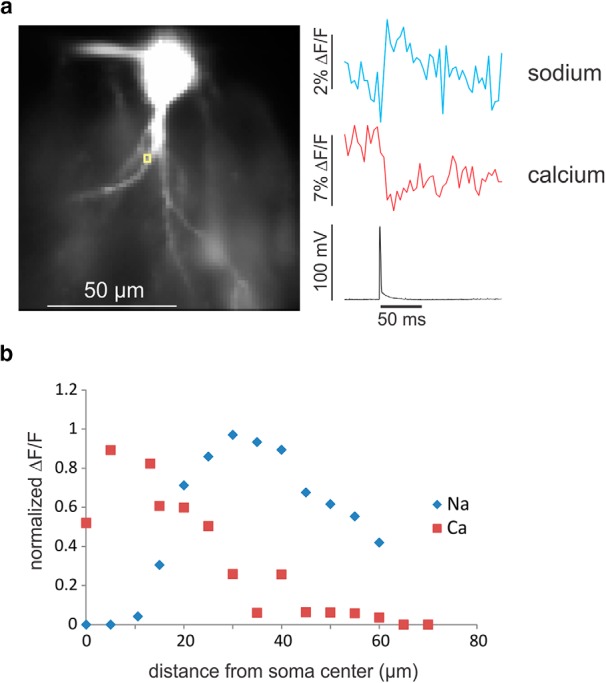
Simultaneous recording of spike evoked sodium and calcium signals from the axon of a CA1 pyramidal neuron. ***a***, Left, pyramidal neuron filled with ANG-2 (200 µM) and bis-fura-2 (200 µM) from a patch electrode on the soma. Intrasomatic current injection evoked a single spike that generated a sharp fluorescence change in each channel at the ROI (yellow box, single pixel) on the axon. The sodium signal appears to rise within one frame, the calcium signal within two frames. There is no delay from the time of the AP. Traces have been corrected for bleaching. ***b***, Plot of the relative ΔF/F of the two signals along the axon, normalized to the peak values (average of 2 cells). These measurements were made with 3 × 3 µm^2^ ROIs to improve the S/N of the measurements. The distributions are clearly different, with the calcium signal peaking in the soma and the sodium signal peaking ∼30 µm from the center of the soma.

We performed additional tests to measure the quality and accuracy of the simultaneous signals. In one experiment, we patched a CA1 pyramidal neuron and evoked a single action potential with this electrode using a short (1 ms) intrasomatic pulse. Figure 4*a* shows the fluorescence changes in the two channels recorded from a single pixel (∼1.1 µm^2^). Both signals rise to peak values within 1–2 frames (4–8 ms). The longer time (in the calcium channel) may reflect the fact that the two channels are not exactly synchronous (2 ms delay) and the spike may have occurred in the middle of the frame. These results show that neither step in the process (indicator–ion reaction or electronic processing in the camera) introduces a significant delay from the time the ion entered the axon. This confirms that the delay in the sodium signal in Figure 3 is physiological.

These signals were measured in a single trial without filtering. In previous experiments on axons (Fleidervish et al., 2010) extensive signal averaging was needed to achieve comparable S/N (see Discussion). Figure 4*b* shows the simultaneously measured spatial distributions of the two signals along the axon. They are clearly different. The calcium signal is largest in the soma and declines with distance along the axon. The sodium signal is very low in the soma and peaks ∼30 µm from the center of the cell body. Because we are measuring the signals from single action potentials (APs) the peak amplitude will not be distorted by diffusion of ions (particularly Na^+^) away from the site of entry (Fleidervish et al., 2010) making the interpretation in terms of ion flux more straightforward. Future experiments will examine the reasons for these different distributions.

**Figure 5.**
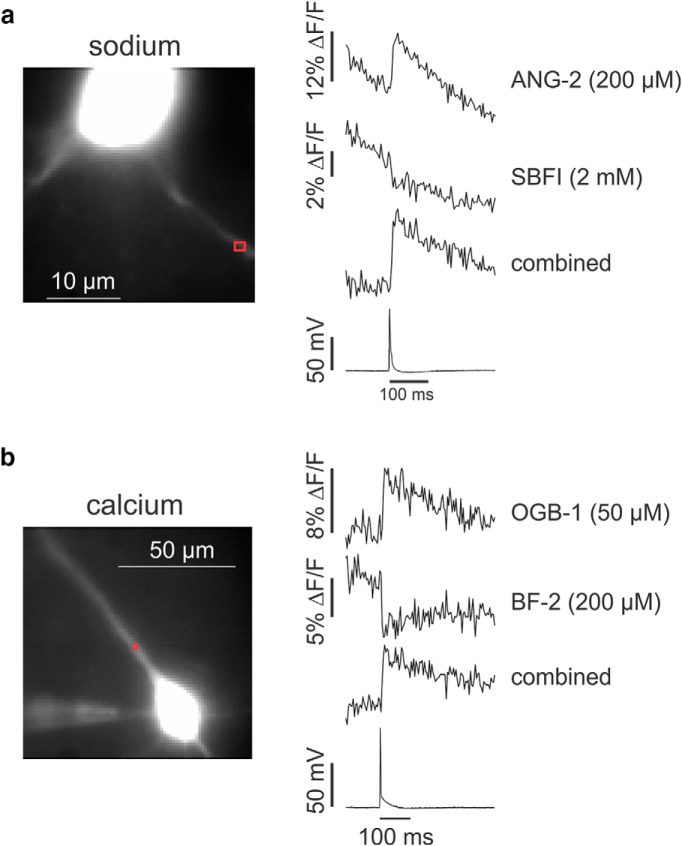
Simultaneous detection of sodium or calcium changes with two different indicators. ***a***, Pyramidal neuron filled with both ANG-2 and SBFI from a patch electrode on the soma. The ANG-2 filter set (Fig. 1*d*) was used with excitation filters centered at 380 and 520 nm. A single AP was evoked with a 2 ms intrasomatic current pulse. ANG-2 and SBFI fluorescence changes in the axon were detected in the two channels. The downward slope indicates strong bleaching due to the high light intensity. The combined trace shows the SBFI trace (1.5×) subtracted from the ANG-2 trace. Most of the bleaching is cancelled and the signal enhanced. Typical result from three cells. ***b***, Similar recordings in the dendrite when two calcium indicators were used. The OGB-1 filter set (Fig. 1*b*) was used with excitatory filters centered at 387 and 470 nm. The combined trace is bis-fura-2 (BF-2; 1.3×) subtracted from the OGB-1 trace. Typical result from four cells.

This technique can be used to simultaneously measure the signals from any two indicators provided their excitation bands are sufficiently separated. For example, Figure 5 shows experiments where neurons were loaded with two different sodium indicators or two different calcium indicators. In Figure 5*a*, spike-evoked sodium signals in the axon were measured simultaneously with ANG-2 and SBFI. The filter set shown in Figure 1*d* could be used because the excitation band for SBFI is close to the excitation band for bis-fura-2. The traces show strong bleaching since the maximum LED intensity was used together with the 1.1 NA 60× lens. Because the signals from the two indicators go in the opposite direction, we could improve the signal by subtracting one trace from the other. This subtraction cancels most of the bleaching and reduces any other common mode noise, like movement artifacts. Figure 5*b* shows a similar experiment measuring the calcium signals in a pyramidal neuron apical dendrite using both bis-fura-2 and OGB-1. The two indicators measured the same response since the exogenous buffering is contributed by both indicators, even when the response is measured from one indicator or the other. There is much less bleaching in this experiment because a 20× lens was used with a much lower NA (0.5) than the 60× lens in Figure 5*a*.

**Figure 6.**
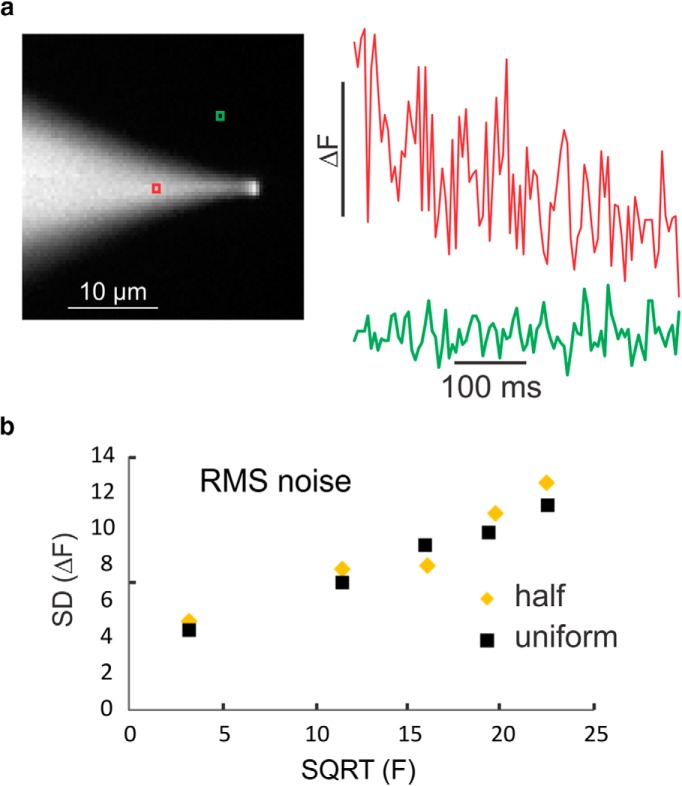
The switching protocol does not introduce additional noise, which is dominated by shot noise. ***a***, Recording of fluorescence levels from two positions in a field containing a pipette filled with ANG-2 while the illumination at 520 nm was switched on and off on alternate frames. Only the signals from the illuminated frames are shown. There clearly is a higher noise level from the position on the pipette. The downward slope is due to bleaching of ANG-2. ***b***, Plot of the SD of the noise level at five different illumination intensities at the red ROI. The yellow diamonds show the results from the condition in ***a*** when only alternate frames were illuminated (“half”). The black squares show the SD when the same measurement was made (only alternate frames) but when the illumination was the same in all frames (“uniform”). There is essentially no difference between the two conditions. Both the diamonds and squares appear to lie along a straight line, the result expected when the system is shot noise limited.

One question we tried to answer was whether the switching protocol introduced any additional noise into the measurement of the optical signals. Figure 6*a* shows an image of a patch pipette containing ANG-2. The two traces show high resolution recordings from the two locations in the image; one from the brighter pipette and the second from the darker background location. The recordings only show the ANG-2 channel after separating the two streams. The other channel was dark because there was no bis-fura-2 in the pipette. The bright channel has more noise, consistent with noise due to light fluctuations. The fluctuations in the signal from the dark region may reflect camera read noise. There is also a downward slope to the recording, which is due to indicator bleaching.

A more quantitative estimate of the source of the noise was obtained by making measurements at different intensities. Figure 6*b* (yellow diamonds) plots the SD of the fluorescence noise of the measurement at the red location against the fluorescence level. The points lie almost along a straight line, which is expected for a “shot noise” limited system. The intercept on the *y*-axis reflects the residual camera noise. We repeated this experiment when the second channel had the same light intensity as the first channel, ie, constant illumination throughout the trial. In this case (black squares), the noise level was essentially the same as the trial when the second channel was dark. These measurements establish that the switching protocol does not degrade the signals and that the limiting noise is shot noise.

## Discussion

In this paper, we describe a system for making simultaneous, high-speed, sensitive sodium and calcium measurements in highly localized regions of neurons. This system as described here uses a RedShirtImaging NeuroCCD-SMQ running at 500 Hz, effectively resulting in a 250 Hz frame rate in each channel. The camera can run faster. We tested the system at 1000 Hz, giving 500 Hz in each channel. Good signals were obtained at this speed, although the noise was larger because of the wider bandwidth. The lower speed (500 Hz) should be adequate for most neurobiologic experiments. The greater S/N in these test experiments compared to previous experiments (Fleidervish et al., 2010) is because of: (1) the significantly higher light intensity of the LEDs, (2) the higher numerical aperture lens, and (3) the greater sensitivity of ANG-2 compared with SBFI. We can quantitatively estimate these improvements. As noted above, at the relevant wavelengths the LED light intensities are ∼9 times higher than the intensities from the arc lamp used previously. The higher NA lens (1.1 compared to 0.9 used previously) yields another factor of 2.2 in intensity at the camera (4th power of the NA ratio). Together these factors provide an improvement of ∼4.4 in the S/N if the noise is shot limited (Fig. 6), because in this case, the S/N scales with square root of the intensity. The improvement from the choice of ANG-2 instead of SBFI depends on the concentrations of the indicators. Typically, we used 2 mM SBFI and 200 µM ANG-2. Using the data from the traces in Figure 5*a* (noise calculated as in Fig. 6) indicates an improvement of ∼1.3 in S/N, yielding a total improvement of ∼6. These calculations assume that only one LED is used with no switching as in the previous experiments (Fleidervish et al., 2010).

Using high-intensity LEDs does cause more indicator bleaching (see Fig. 5*a*) and photodynamic damage. The bleaching can be corrected with software, for example, by recording a trace without physiological stimulation and subtracting it from the stimulated trace after normalizing intensities at the beginning of the traces. Greater photodynamic damage, which is often a problem in live imaging experiments, is harder to avoid. Limiting the exposure time of the preparation, using as low concentrations of indicator as possible, and controlling the intensity are all important. In some cases, photodynamic damage can be reduced by replacing oxygen with nitrogen in the ACSF. One advantage of LEDs, compared with arc lamps, is that their intensity can be easily controlled by changing the current. This allows us to adjust the total integrated light per frame in each channel either by controlling the pulse amplitude or the pulse width. This adjustment is valuable for achieving optimal S/N in each channel. We found it more convenient to adjust the pulse width, but either approach will work.

Figure 3 shows that we could detect signals without averaging from submicron regions in the dendrites. In principle, this resolution should be good enough to detect signals from individual spines. Although we have some candidate signals (not shown), light scattering in the tissue and out of focus elements made it difficult to resolve spine signals cleanly. Using cultured slices, which flatten in time or searching for spines close to the surface of an acute slice may make these experiments easier, as has been done in some voltage-sensitive dye experiments (Popovic et al., 2014). Two-photon microscopy can clearly resolve spines and sodium signals from spines have been reported (Rose and Konnerth, 2001). However, those signals were summations over many spines and were recorded at low time resolution (4–8 Hz). Other groups have reported failure to detect spine sodium signals from single stimuli using SBFI (Araya et al., 2007).

We used two different combinations of indicators: SBFI/OGB-1 and ANG-2/bis-fura-2. Because OGB-1 is more sensitive than bis-fura-2 for detecting small [Ca^2+^]_i_ changes, the first combination is preferred where detecting small [Ca^2+^]_i_ changes is the main goal. Similarly, the second combination is preferred when detecting small [Na^+^]_i_ changes is desired, although other considerations, such as photodynamic damage, may enter into the choice. If possible, both combinations should be tested for each experimental condition.

Different approaches to simultaneous imaging have been used by other investigators. Many experiments with two-photon imaging excite at one wavelength and simultaneously record from red and green channels, where one channel provides an intensity reference for the dynamic signals in the other channel (Yasuda et al., 2003). In one case, this method was used to detect sodium and calcium signals in axons (Bender and Trussell, 2009). They measured signals using CoroNa green (sodium) and X-rhod-5F (calcium). However, the S/N of their recordings does not appear to be as good as in our records. An analogous approach using two CCD cameras to detect the emitted fluorescence from voltage and calcium indicators was used to detect simultaneous physiological signals from neurons in brain slices (Vogt et al., 2011). Using a technique related to ours, two groups used LED switching to excite and record calcium and voltage signals from the intact isolated heart (Lee et al., 2011) and cardiac myocytes (Scull et al., 2012).

## Synthesis

The decision was a result of the Reviewing Editor Michael Hausser and the peer reviewers coming together and discussing their recommendations until a consensus was reached. A fact-based synthesis statement explaining their decision and outlining what is needed to prepare a revision is listed below. The following reviewers agreed to reveal their identity: Dejan Zecevic, Thomas Knopfel

This paper describes a novel strategy for near-simultaneous detection of Ca2+ and Na+ intracellular concentration transients from axonal and dendritic processes of individual neurons. The authors combined a low molecular weight calcium indicator with a sodium indicator and use two high intensity LEDs at two excitation wavelengths on an upright epifluorescent microscope to image changes in calcium and voltage of rat hippocampal neurons in brain slices. This is a novel and potentially very useful approach that will allow investigators to address questions that are inaccessible using currently available methods.

Detailed comments:

1. The authors found that high intensity LED (light emitting diodes) light sources, a low noise, high speed CCD camera, and high numerical aperture objectives all improved the signals. While this is expected, no formal evidence is provided to support this statement. It would be helpful if the authors could provide a direct comparison of the S/N available "before and after".

2. The elimination of crosstalk is crucial for this approach, which combines two indicators. For example, do the traces labelled "sodium" represent only ANG-2 fluorescence? The authors should discuss this issue more extensively, and ideally provide controls to show that the traces labelled calcium and sodium are indeed calcium and sodium signals, respectively, e.g. the "sodium" signal that remains when applying a calcium channel blocker.

3. The authors "designed filters" but only give partial information allowing other researchers to replicate the approach. A more detailed preparation of Fig 1 and a table of filter combinations for all dye combinations tested would be helpful. For example, Figure 1 currently only shows excitation and emission spectra of SBFI and OGB-1 - but ANG-2 is used in all of the rest of the figures (no experimental data is provided for SBFI). At the very least, the authors need to also provide the spectra for ANG-2.

4. The authors state that "using high intensity LEDs does cause more indicator bleaching and photodynamic damage. The bleaching can be corrected with software". Please provide more details about how the bleaching correction was carried out. Was bleaching correction carried out on the figures presented in the manuscript? This should be stated.

5. The text in the Abstract and Significance would benefit from a clear example of a question that can be addressed by the described approach while it cannot be answered in any other way. The statement "...measurements of both [Ca2+]i and [Na+]i changes in the same preparation may provide more information than separate measurements" is too vague.

6. Figures 3 and 4: please include an absolute Y-scale as a fractional change in light intensity. This would help the reader assess the available sensitivity better.

7. In Figure 3 and 4a, please color the imaging traces to match Figure 4b (i.e. sodium blue, calcium red).

8. The authors compared two sodium indicators, but remained inconclusive on whether the one generally works better than the other or which experimental conditions would influence the choice. It would be helpful to provide more information here to settle the issue. Note that only experimental data for ANG-2 is provided in the figures - does this mean that SBFI was clearly inferior for all of these experiments shown in the figures?

9. The authors point out that sodium imaging would be valuable to image activation of AMPA receptors. To reference this idea, Scelfo et al (J Neurophysiology. 2003 89:2555-63) should be cited.

10. Since this is a methodological paper, please provide a statement that any additional technical details, and custom software (e.g. SCAN - was this written by the authors? Please clarify), can be obtained on request.
